# Adipose tissue immunity and cancer

**DOI:** 10.3389/fphys.2013.00275

**Published:** 2013-10-02

**Authors:** Victoria Catalán, Javier Gómez-Ambrosi, Amaia Rodríguez, Gema Frühbeck

**Affiliations:** ^1^Metabolic Research Laboratory, Clínica Universidad de NavarraPamplona, Spain; ^2^CIBER Fisiopatología de la Obesidad y Nutrición, Instituto de Salud Carlos IIIPamplona, Spain; ^3^Department of Endocrinology and Nutrition, Clínica Universidad de NavarraPamplona, Spain

**Keywords:** adipose tissue, inflammation, immune cells, adipokines, angiogenesis, hypoxia, macrophages, tumor growth

## Abstract

Inflammation and altered immune response are important components of obesity and contribute greatly to the promotion of obesity-related metabolic complications, especially cancer development. Adipose tissue expansion is associated with increased infiltration of various types of immune cells from both the innate and adaptive immune systems. Thus, adipocytes and infiltrating immune cells secrete pro-inflammatory adipokines and cytokines providing a microenvironment favorable for tumor growth. Accumulation of B and T cells in adipose tissue precedes macrophage infiltration causing a chronic low-grade inflammation. Phenotypic switching toward M1 macrophages and Th1 T cells constitutes an important mechanism described in the obese state correlating with increased tumor growth risk. Other possible synergic mechanisms causing a dysfunctional adipose tissue include fatty acid-induced inflammation, oxidative stress, endoplasmic reticulum stress, and hypoxia. Recent investigations have started to unravel the intricacy of the cross-talk between tumor cell/immune cell/adipocyte. In this sense, future therapies should take into account the combination of anti-inflammatory approaches that target the tumor microenvironment with more sophisticated and selective anti-tumoral drugs.

## Introduction

The incidence of obesity and its associated disorders is increasing at an accelerating and alarming rate worldwide (Flegal et al., 2012; Frühbeck et al., 2013). Relative to normal weight, obesity is associated with significantly higher all-cause mortality (Frühbeck, 2010; Flegal et al., 2013). Body mass index (BMI) represents the most used diagnostic tool in the current classification system of obesity, frequently used as an indicator of body fat percentage (BF). The controversy in studies (Hughes, 2013) arises in part because a wide variety of BMI cutoffs for normal weight has been applied to correlate with mortality which can yield quite diverse findings. Furthermore, in spite of its wide use, BMI is only a surrogate measure of body fat and does not provide an accurate measure of body composition (Frühbeck, 2012; Gómez-Ambrosi et al., 2012). Noteworthy, obesity is defined as a surplus of body fat accumulation, with the excess of adipose tissue really being a well-established metabolic risk factor for the development of obesity-related comorbidities such as insulin resistance, type 2 diabetes (T2D), cardiovascular diseases and some common cancers (Bray, 2004; Kahn et al., 2006a; Van Gaal et al., 2006; Renehan et al., 2008; Bardou et al., 2013).

Results from epidemiological studies indicate that overweight and obesity contribute to the increased incidence and/or death from quite diverse types of cancers, including colon, breast (in postmenopausal women), endometrium, kidney (renal cell), esophagus (adenocarcinoma), stomach, pancreas, gallbladder and liver, among others (Calle and Kaaks, 2004). The mechanisms linking excess of adiposity and cancer are unclear but the obesity-associated low-grade chronic inflammation is widely accepted as an important factor in cancer pathogenesis (Catalán et al., 2011d; Hursting and Dunlap, 2013). Chronic hyperinsulinaemia as well as the alterations in the production of peptide and steroid hormones associated to obesity are other postulated mechanisms involved in cancer development (Calle and Thun, 2004). Particular attention is placed on the pro-inflammatory microenvironment associated with the obese state (Catalán et al., 2011d; Ribeiro et al., 2012; Hursting and Dunlap, 2013), specifically highlighting the involvement of obesity-associated hormones/growth factors in the cross-talk between macrophages, adipocytes, and epithelial cells in many cancers. Among the various pathophysiological mechanisms postulated to explain the link between obesity and cancer, the dysfunctional adipose tissue may be a unifying and underlying factor (van Kruijsdijk et al., 2009). Understanding the contribution of obesity to growth factor signaling and chronic inflammation provides mechanistic targets for disrupting the obesity-cancer link (Harvey et al., 2011).

In this regard, obesity prevention is a major part of several evidence-based cancer prevention guidelines (Kushi et al., 2012). Recent studies exploring the effect of weight loss, suggest that severe caloric restriction in humans may confer protection against invasive breast cancer (Michels and Ekbom, 2004). This protective effect includes reductions in the initiation and progression of spontaneous tumors in several tissues (Longo and Fontana, 2010). Moreover, the association between obesity and cancer is consistent with data from animal models showing that caloric restriction decreases spontaneous and carcinogen-induced tumor incidence (Dunn et al., 1997; Yun et al., 2013). Both bariatric surgery and short-term intentional weight loss have been shown to improve insulin sensitivity and inflammatory state, which have been postulated to contribute to the relationship between obesity and cancer (Sjöström et al., 2007; Cummings et al., 2012).

## The importance of obesity-induced chronic inflammation

Adipocytes, the principal cellular component of adipose tissue, are surrounded by connective tissue comprising macrophages, fibroblasts, preadipocytes, and various cell types included in the stromovascular fraction (Hausman et al., 2001; Nishimura et al., 2007; Cinti, 2012). Although adipocytes have been considered primarily as fat-storage depots, in recent years, it has become clear that together with other metabolically active organs, adipose tissue is a dynamic endocrine system key in the regulation of whole body energy homeostasis (Frühbeck et al., 2001a; Ahima, 2006; Sáinz et al., 2009). Indeed, mature adipocytes are involved in endocrine, paracrine, and autocrine regulatory processes (Ahima and Flier, 2000) through the secretion of large number of cytokines, hormones and other inflammatory markers, collectively termed adipokines (Lago et al., 2007, 2009; Lancha et al., 2012). In addition to playing key roles in the regulation of the lipid and glucose homeostasis, adipokines modify physiological processes, such as hematopoiesis, reproduction, and feeding behavior, being also involved in the genesis of the multiple pathologies associated with an increased fat mass including cancer development (Rajala and Scherer, 2003). However, adipose tissue not only secretes adipokines but also functions as a target of these pro-inflammatory mediators, expressing a wide variety of receptors for cytokines, chemokines, complement factors, and growth factors (Frühbeck, 2006a; Fruhbeck, 2006b; Schäffler and Schölmerich, 2010).

The connection between inflammation and diabetes was suggested more than a century ago (Williamson, 1901), but the evidence that inflammation is an important mediator in the development of insulin resistance came recently. It was described that the administration of tumor necrosis factor-α (TNF-α) led to increased serum glucose concentrations (Feingold et al., 1989). The first study that established the concept of obesity-induced adipose tissue inflammation was conducted by Hotamisligil et al. (1993), demonstrating that the pro-inflammatory cytokine TNF-α mediate insulin resistance in many experimental models of obesity. Importantly, the development of adipose tissue has been associated with increased plasma levels of well-known inflammatory and acute phase proteins such as C-reactive protein, interleukin (IL)-6, IL-8, serum amyloid A (SAA) and monocyte chemotactic protein (MCP)-1 in patients and different animal models of obesity (Frühbeck et al., 1995; Wellen and Hotamisligil, 2003; Frühbeck, 2005; Gómez-Ambrosi et al., 2006; Kahn et al., 2006b; Kim et al., 2006; Catalán et al., 2007, 2008), whereas production of the anti-inflammatory and insulin-sensitizing adipokine adiponectin is reduced with increasing body weight (Kadowaki et al., 2006). In obesity, the activation of the c-Jun N-terminal kinase (JNK) and nuclear factor κ B (NF-κ B) transduction signals is key in the inflammation process of adipose tissue and these pathways could interact with insulin signaling via serine/threonine inhibitory phosphorylation of IRS (Bastard et al., 2006; Gil et al., 2007). Genetic or pharmacological manipulations of these different effectors of the inflammatory response modulate insulin sensitivity in different animal models.

Recent data suggest that stromovascular cells also contribute to the secretion of inflammatory adipokines. In this sense, the infiltration of adipose tissue by immune cells is a feature of obesity, with adipose tissue macrophage (ATM) accumulation being directly proportional to measures of adiposity in both mice and humans (Weisberg et al., 2003). This evidences a role of adipose tissue as part of the innate immune system.

## Adipose tissue inflammation, a microenvironment for tumorigenesis

Analogously to adipose tissue, the tumor microenvironment is composed by multiple cell types including epithelial cells, fibroblasts, mast cells, and cells of the innate and adaptive immune system that favor a pro-inflammatory and pro-tumorigenic environment (Harvey et al., 2011). These inflammatory cells secrete cytokines, growth factors, metalloproteinases, and reactive oxygen species, which can induce DNA damage and chromosomal instability, thereby favoring carcinogenesis (Khasawneh et al., 2009). The abundance of leukocytes in neoplasic tissue was crucial to establish the link between chronic inflammation and cancer development (Virchow, 1863). Now, inflammation is a well-known hallmark of cancer, and growing evidence continues to indicate that chronic inflammation is associated with increased cancer risk (Aggarwal and Gehlot, 2009).

The expanded adipose tissue constitutes an important initiator of the microenvironment favorable for tumor development (Catalán et al., 2011d) due to its ability to produce and secrete inflammatory cytokines by adipocytes or infiltrating macrophages (Xu et al., 2003). Noteworthy, novel adipokines [lipocalin-2 (LCN-2), osteopontin (OPN) and YKL40] related to inflammation and insulin resistance with emerging roles in tumor development have been recently described to be increased in adipose tissue from patients with colon cancer (Catalán et al., 2011d).

In this line, periprostatic adipose tissue of obese subjects shows a dysregulated expression of genes encoding molecules involved in inflammatory processes including antigen presentation, B cell development, and T helper cell differentiation. Moreover, subjects with prostate cancer display an altered profile of genes with great impact on immunity and inflammation in their periprostatic adipose tissue (Ribeiro et al., 2012). The up-regulation of complement factor H and its receptor in periprostatic adipose tissue from patients with prostate cancer has been also described, suggesting an inhibitory modulation of the complement activity in prostate tumor cells and evasion to attack. Other altered molecules include the B lymphocyte antigen CD20 encoded by the *MS4A1* gene with a functional role in B-cell activation and *FFAR2* that encodes a protein reported to modulate the differentiation and/or activation of leukocytes (Ribeiro et al., 2012). This observation highlights the bi-directional interactions between periprostatic adipose tissue and tumor cells, which influence adipose tissue function and may influence prostate cancer progression inducing an environment favorable to cancer progression.

Clusters of enlarged adipocytes become distant from the vasculature in expanding adipose tissue leading to local areas of hypoxia and eventually necrosis. The reduction in oxygen pressure associated with adipose tissue hypoxia is considered to underlie the inflammatory response (Trayhurn et al., 2008; Ye, 2009; Trayhurn, 2013). The master regulator of oxygen homeostasis is the hypoxia-inducible factor (HIF)-1α. HIF-1α is increased in the adipose tissue of obese patients and its expression is reduced after surgery-induced weight loss (Cancello et al., 2005). It is well-documented that HIF-1α also influences both the innate and the adaptive immunity regulating functions of myeloid cells, neutrophils, macrophages, mast cells, dendritic cells, natural killer cells and lymphocytes (Eltzschig and Carmeliet, 2011). Similarly to what takes place in tumor tissue, adipose tissue hypoxia is related to the presence of macrophages, which migrate to the hypoxic regions and alter their expression profile increasing inflammatory events (Fujisaka et al., 2013). Hypoxia activation is a critical microenvironmental factor during tumor progression with oxygen concentrations in solid tumors being frequently reduced compared with normal tissues (Semenza, 2003; Jiang et al., 2011). HIF-1α and HIF-2α are overexpressed in certain solid tumors (Zhong et al., 1999; Talks et al., 2000), with these elevated levels being associated with cancer-related death in specific tumoral types of the brain (oligodendroglioma), breast, cervix, oropharynx, ovary, and uterus (endometrial) (Semenza, 2003). HIF-2α is also strongly expressed by subsets of tumor-associated macrophages, sometimes in the absence of expression in any tumor cell (Talks et al., 2000). Overall, hypoxia has effects on the function of adipocytes and appears to be an important factor in adipose tissue dysfunction in obesity increasing the risk of cancer development.

Moreover, hypoxia is a primary physiological signal for angiogenesis (growth of blood vessels) in both physiological and pathological conditions. Angiogenesis is a physiological response that regulates adipogenesis representing a hallmark of tumor growth (Hanahan and Folkman, 1996; Carmeliet and Jain, 2000; Cao, 2007). Adipocytes seem regulate angiogenesis both by cell to cell contact and by adipokine secretion (Cao, 2007; Lemoine et al., 2013). In this regard, many cytokines produced by adipose tissue show angiogenic activities such as leptin, TNF-α, IL-6, IL-8, vascular endothelial growth factor (VEGF) and tumor growth factor β (TGF-β) (Ferrara and Kerbel, 2005; Ye, 2009; Gómez-Ambrosi et al., 2010).

The blocking of tumor angiogenesis as an anticancer strategy has shown desirable results across multiple tumor types (Folkman, 1971; Schneider et al., 2012). The standard chemotherapy usually results in partial or total resistance after different cycles of treatment (Kerbel, 1997). Based on the hypothesis that endothelial cells have a normal complement of chromosomes and a relative genetic stability, the use of inhibitors of angiogenesis may avoid acquired drug resistance (Kerbel, 1997). Current pharmacotherapeutic options for treating obesity and related metabolic disorders remain limited and ineffective. Emerging evidence shows that modulation of angiogenesis is a possible therapeutic intervention to impair the development of obesity by regulating the growth and remodeling of the adipose tissue vasculature (Rupnick et al., 2002; Cao, 2010). Adipose tissue growth is angiogenesis-dependent (Rupnick et al., 2002) and the modulation of angiogenesis appears to have the potential to impair the development of obesity (Lijnen, 2008). Studies in mice have shown that the administration of anti-angiogenic agents prevents diet-induced or genetic obesity (Brakenhielm et al., 2004a). Genetically obese mice treated with different angiogenesis inhibitors such as TNP-470, angiostatin, endostatin, Bay-129566, a matrix metalloproteinase inhibitor, or thalidomide showed reduced body and adipose tissue weights as well as increased apoptosis in the adipose tissue compared with control mice (Rupnick et al., 2002). In this regard, targeting a proapoptotic peptide to prohibitin in the adipose vasculature caused ablation of white fat in both, diet-induced and age-related obesity (Kolonin et al., 2004). Recently, the antiangiogenic treatment blocking VEGFR2 by antibodies but not of VEGFR1 has been described to limit adipose tissue expansion (Tam et al., 2009). To evaluate the effects of the different antiangiogenic agents characterized in the cancer field in obesity models *in vivo* may be an attractive target to limit adipose tissue expansion. However, a too strong inhibition of adipose tissue expansion by impairing angiogenesis may lead to ectopic lipid storage, increased inflammation, and further deterioration of systemic insulin sensitivity (Sun et al., 2012; Lemoine et al., 2013). Moreover, adipose tissue development is a multifactorial process and it is unlikely that a single angiogenesis inhibitor will allow reduction of obesity without associated side effects (Lijnen, 2008). Thus, blocking the capacity for angiogenesis may have different outcomes, depending on the stage of obesity.

## Immune cell types present in expanded adipose tissue

**Figure 1:**
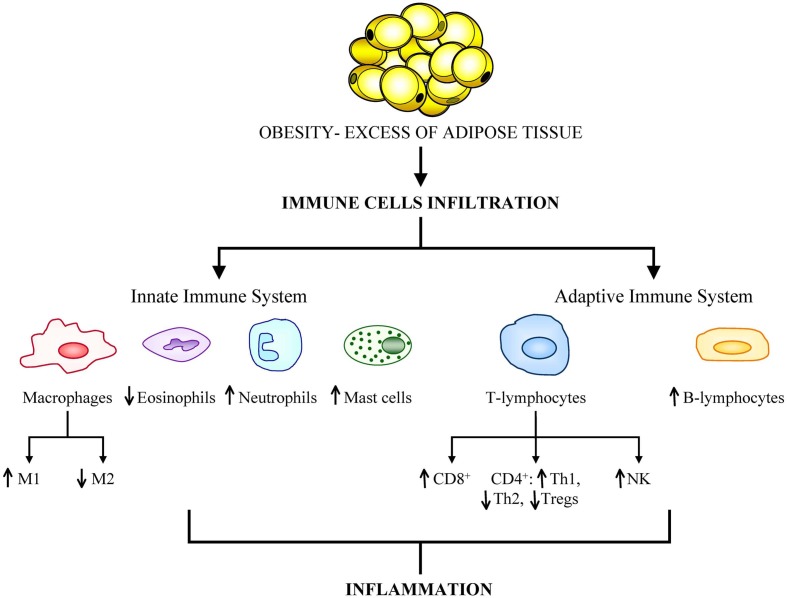
**Obesity is associated with a great infiltration of cells from both the innate and adaptive immune systems.** The aberrant population expansion of these cells is related to the onset of obesity-related comorbidities, primarily cancer development.

In cases of severe obesity, adipose tissue can constitute up to 50–60% of the total body mass being the expanded adipose tissue a largely uncharacterized immunological organ with distinct subpopulations of cells of the immune system (Kanneganti and Dixit, 2012). Furthermore, excess of body fat is accompanied by altered immune cell function and different expression profile of genes related to immunity in obese human subjects compared with healthy-weight individuals (Gómez-Ambrosi et al., 2004). Discrepancies in leukocyte number, subset, and activity of monocytes between lean and obese individuals have been reported (Nieman et al., 1999). Adipose tissue has been shown to exhibit a dynamic infiltration by innate and adaptive cells during the onset of insulin resistance and diet-induced obesity (Duffaut et al., 2009). The observation of infiltrated macrophages in the adipose tissue of obese patients prompted an increased interest in the interplay between immune cells and metabolism. Recent studies have revealed a growing list of immune cell types (including macrophages, lymphocytes, mast cells, eosinophils neutrophils and foam cells) that infiltrate adipose tissue and have potential roles in insulin resistance (Olefsky and Glass, 2010; Dalmas et al., 2011; Wu et al., 2011; Shapiro et al., 2013) (Figure 1).

The role of adaptive immune cells in obesity-induced adipose tissue inflammation has been less characterized than that of innate immune cells. Based on studies in mouse models, lymphocyte infiltration in adipose tissue might occur in a chronological sequence. B and T lymphocytes are recruited during early obesity-induced inflammation by preadipocytes or chemotactic adipokines like CCL5, CXCL5, CXCL12, or CCL20. Furthermore, the cytokines derived from Th lymphocytes reportedly modulate macrophage phenotype switching, which is directly linked to insulin resistance (Sell et al., 2012).

To explain the chronological order of how immune cells infiltrate adipose tissue in obesity, it has been proposed that T cells may stimulate preadipocytes to induce the recruitment of macrophages via chemotactic factors such as MCP-1, shedding new light on the importance of chemotaxis in this scenario (Kintscher et al., 2008).

## Innate immune system in adipose tissue

Macrophages and monocytes are representative of the innate immune system and represent a large proportion of the stromovascular cell fraction in adipose tissue. Several cell types of the innate immune system are involved in the development of adipose tissue inflammation and the most studied cell type among these is the ATM (Kalupahana et al., 2012). Neutrophils and mast cells, also members of the innate immune system have been also implicated in promoting inflammation and insulin resistance during obesity, whereas eosinophils and myeloid-derived suppressor cells have been suggested to play a protective role (Wu and Van Kaer, 2013).

### Monocytes and macrophages in adipose tissue

The majority of macrophages found in the adipose tissue of diet-induced obese mice are originated from blood monocytes (Weisberg et al., 2003; Dalmas et al., 2011). Monocytes are a heterogeneous cell population that differ in their migration and cell fate properties (Saha and Geissmann, 2011). The phenotype of macrophages depends on the subset of monocytes upon arrival at target tissues being probably determined by the local microenvironment (Dalmas et al., 2011). The number of resident macrophages present in adipose tissue was found to correlate positively with obesity in various mouse models and in human adipose tissue (Weisberg et al., 2003; Xu et al., 2003). Thus, it is possible to speculate that macrophages might be involved in the growth of the fat mass in a similar manner to that described in tumors (Curat et al., 2004).

Based on their cytokine profile secretion and cell surface markers, ATMs are classified into two main types: the “classical” macrophages named M1 in contrast to the “alternatively activated” M2. M1 macrophages are the first line of defense against intracellular pathogens with high microbicidal activity and are classically stimulated by interferon (IFN)-γ or by lipopolysaccharide (LPS). M1 induce the secretion of inflammatory cytokines (IL-1, IL-6, TNF-α, MCP1) and reactive oxygen species, and nitric oxide (NO) through the stimulation of inducible NO synthase (iNOS) (Lumeng et al., 2008). Alternative activation, resulting from induction by the Th2 cytokines interleukin IL-4 and IL-13 (Gordon, 2003) is associated with tissue repair and humoral immunity producing immunosuppressive factors, such as IL-10, IL-1Ra, and arginase (Gordon and Taylor, 2005). Obesity induces a phenotypic switch from an anti-inflammatory M2-polarized state to a pro-inflammatory M1 state (Lumeng et al., 2007). The importance of the M1/M2 ratio has been reported in macrophage-specific *Pparg-*deficient mice that show impaired alternative macrophage activation, increased development of obesity and adipose tissue inflammation as well as glucose intolerance (Odegaard et al., 2007). The identification of the signaling pathways that control macrophage polarization in expanding adipose tissue remains a challenging issue. In this sense, it has been described that the local hypoxia in expanding adipose tissue may promote the M2 to M1 switching (Ye and McGuinness, 2013). Moreover, a recent study in *Trib1*-deficient mice has shown a severe reduction of M2-like macrophages in adipose tissue highlighting the contribution of Trib1 for adipose tissue homeostasis by controlling the differentiation of tissue-resident M2-like macrophages (Satoh et al., 2013).

### Involvement of neutrophils, eosinophils, and mast cells in obesity

The notion that a transient “acute inflammatory infiltrate” precedes the “chronic inflammatory infiltrate” in obesity and that neutrophils play a key role (Wagner and Roth, 2000) producing chemokines and cytokines, thereby facilitating macrophage infiltration has been proposed (Talukdar et al., 2012). In this line, adipose tissue neutrophils could have a role in initiating the inflammatory cascade in response to obesity based on the fact that mice fed with a high-fat diet show an increase in neutrophil recruitment to adipose tissue peaking at 3–7 days and subsiding thereafter (Elgazar-Carmon et al., 2008). The treatment of hepatocytes with neutrophil elastase causes cellular insulin resistance while deletion of neutrophil elastase in obese mice leads to reduced inflammation (Talukdar et al., 2012).

Although eosinophils are associated with allergic diseases and helmintic infections (Rothenberg and Hogan, 2006), the biologic role of these cells in adipose tissue remains incompletely defined (Maizels and Allen, 2011). It has been shown that eosinophils are the main source of IL-4 and IL-13 in white adipose tissues of mice, and, in their absence, M2 macrophages are greatly attenuated (Wu et al., 2011). Moreover, in the absence of eosinophils, mice which were fed a high-fat diet develop increased body fat and insulin resistance (Wu et al., 2011). The promotion of eosinophil responses can protect against metabolic syndrome (Wu et al., 2011).

Mast cells, like macrophages, are inflammatory cells, but the exact mechanisms of mast cells in the pathogenesis of obesity are not fully understood. In this regard, increased mast cells in adipose tissue from obese subjects compared with those of lean subjects have been reported. Obese subjects also had significantly higher tryptase concentrations in their serum than lean individuals. Mast cells may contribute to inflammation through the secretion of IL-6 and IFN-γ (Stienstra et al., 2011). Moreover, mast cell number is related to fibrosis, macrophage inflammation and endothelial activation of adipose tissue in human obesity (Divoux et al., 2012). These observations suggest a possible association between mast cells and obesity-associated inflammation (Liu et al., 2009; Zhang and Shi, 2012).

## Adaptive immune system in adipose tissue

Recent advances in the field of adipose tissue biology reveal a prominent role of different types of lymphocytes (T-lymphocytes, B-lymphocytes, and natural-killer cells) in adipose tissue inflammation depending on the obese state in parallel to macrophages (Sell and Eckel, 2010).

### T-lymphocytes in adipose tissue

CD4^+^ T cells along with CD8^+^ T cells constitute the majority of T-lymphocytes. Experimental data suggest that T-lymphocytes might play a role in the development of insulin resistance during obesity. In this sense, T-lymphocytes are described in visceral and subcutaneous adipose tissue of obese mice and humans (Bornstein et al., 2000) but the role of different subtypes of lymphocytes, CD4^+^, and CD8^+^ cells, in adipose tissue inflammation remains largely unexplored. The increase in the number of T cells in adipose tissue from diet-induced obesity mice is gender-dependent, with higher numbers of T cells in obese males than in females or lean males (Wu et al., 2007). Based on studies in mouse models, lymphocyte infiltration in adipose tissue might occur in a chronological sequence with T lymphocytes being recruited during early obesity-induced inflammation by chemokines like RANTES, a T-cell specific chemokine also known as CCL5 (Sell et al., 2012). In this regard, the expression of RANTES and its respective receptor CCR5 in visceral adipose tissue of morbidly obese patients have been described (Wu et al., 2007).

CD4^+^ T cells are crucial in achieving a regulated effective immune response to pathogens. In adipose tissue, CD4^+^ T cells are mainly classified into the classical T-helper 1 (Th1) and T-helper 2 (Th2) although new subsets have been identified including T-helper 17 (Th17), induced T-regulatory cells (iTreg), and the regulatory type 1 cells (Tr1), among others (Luckheeram et al., 2012). The roles for CD4^+^ T lymphocytes in adipose tissue are related to the regulation of body weight, adipocyte hypertrophy, insulin-resistance, and glucose tolerance. Thus, CD4^+^ cells are key in the control of disease progression in diet-induced obesity (Winer et al., 2009). Th1 cells show a pro-inflammatory profile, secreting IFN-γ, which elicits the production of macrophage mediators, induces leukocyte adhesion molecules and chemokines, as well as increases antigen-presenting capacity by macrophages and endothelial cells (Geng and Hansson, 1992; Tellides et al., 2000). Interestingly, T cells extracted from fat tissue of obese mice and stimulated *in vitro* produced higher amounts of IFN-γ than those extracted from lean animals. This finding suggests that obesity primes T cells from adipose tissue toward a Th1 switch (Rocha et al., 2008). Winer *et al*. (Winer et al., 2009) reported that the increase of CD4^+^ T cells with obesity in mice is largely due to the accumulation of IFNγ produced by Th1 cells. The elevated levels of IFNγ also contribute to the classical activation of adipose tissue macrophages, resulting in increased inflammation in adipose tissue. On the other hand, Th2 are anti-inflammatory cells and are a source of IL-4 and IL-13. In this regard, T cells may orchestrate an inflammatory cascade, depending on the set of cytokines they predominantly produce (Hansson and Libby, 2006). A dramatic increase in the number of Th1 cells has been described in diet-induced obesity states, whereas the number of Th2 cells remained unchanged (Sell and Eckel, 2010).

T regulatory (Treg) cells are a small subset of T lymphocytes constituting normally 5–20% of the CD4^+^ compartment. Tregs are critical in the defense against inappropriate immune responses such as inflammation and tumorigenesis (Sakaguchi et al., 2008) because they control the behavior of other T cell populations and influence the activities of the innate immune system cells (Maloy et al., 2003). Treg cells regulate the activities of macrophages and adipocytes probably secreting IL-10, given their association with improved insulin sensitivity in both rodents and humans (Scarpelli et al., 2006). It has been recently described that the accumulation of Tregs in visceral adipose tissue is mediated by the nuclear receptor peroxisome proliferator-activated receptor (PPAR)-γ (Cipolletta et al., 2012). PPAR-γ tended to impose the transcriptional characteristics of visceral adipose tissue Tregs on naïve CD4^+^ T cells (Cipolletta et al., 2012). Tregs may be regulated by local hypoxia, increased adipocyte death and adipocyte stress (Feuerer et al., 2009). The diminished Treg cells in obesity could promote the infiltration of macrophages in adipose tissue and, thereby, increase the production of inflammatory cytokines.

CD8^+^ T cells are involved in the initiation and propagation of inflammatory cascades in obese adipose tissue (Nishimura et al., 2009). CD8^+^ cells are required for adipose tissue inflammation and have major roles in macrophage differentiation, activation and migration (Nishimura et al., 2009). A study in mice reported mainly CD8^+^ lymphocyte infiltration in hypoxic areas of epididymal adipose tissue in mice fed a high-fat diet, whereas the numbers of CD4^+^ and regulatory T cells were reduced (Rausch et al., 2008). The infiltration by CD8^+^ T cells precedes the recruitment of macrophages. Indeed, immunological and genetic depletion of CD8^+^ T cells lowered macrophage infiltration and adipose tissue inflammation as well as ameliorated systemic insulin resistance (Rausch et al., 2008). Another study also demonstrates an early T lymphocyte infiltration during the development of insulin resistance in a mouse model of high fat diet-induced obesity as well as a correlation of T cells with waist circumference in diabetic patients (Kintscher et al., 2008), highlighting the association of insulin resistance with adipose tissue lymphocyte infiltration. Oppositely, most of these cells were CD4^+^ with only a few CD8^+^ cells.

Recent studies have focused on another regulatory T cell subset, natural killer T (NKT) cells, in the development of obesity-associated inflammation and comorbidities (Lukens and Kanneganti, 2012; Lynch et al., 2012). NKT cells are abundant in metabolically active organs such as liver and adipose tissue (Emoto and Kaufmann, 2003; Lynch et al., 2009) and show the capacity to produce a variety of both pro- and anti-inflammatory cytokines (Wu and Van Kaer, 2013). NKT cells exert their effects in the development of inflammation and metabolic diseases in response to nutritional lipid excess (Wu and Van Kaer, 2013).

### B-lymphocyte accumulation in dysfunctional adipose tissue

A fundamental pathogenic role for B cells in the development of metabolic abnormalities has been described (Winer et al., 2011; DeFuria et al., 2013). In mice, B-lymphocytes accumulate in adipose tissue before T cells, shortly after the initiation of a high-fat diet (Duffaut et al., 2009). The early recruitment of B cells promotes T cell activation and pro-inflammatory cytokine production, which potentiates M1 macrophage polarization and insulin resistance (Winer et al., 2011).

Moreover, an impaired function of toll-like receptors in B cells from patients with T2D that increases inflammation by the elevation of pro-inflammatory IL-8 and lack of anti-inflammatory/protective IL-10 production has been described (Jagannathan et al., 2010).

## Adipokine dysregulation and cancer

A growing body of evidence suggests that the inflammatory milieu of the obese state is linked to the development of cancer through different mechanisms (Grivennikov et al., 2010). Infiltrating immune cells in adipose tissue regulates the local immune response, inducing increased levels of pro-inflammatory cytokines and adipokines and providing a major link to the obesity-associated tumor development (van Kruijsdijk et al., 2009). Critical molecules involved in the promotion of tumor cell proliferation include inflammatory transcription factors [such as NF-κ B and signal transducer and activator of transcription 3 (STAT3)], adipokines (leptin and adiponectin) as well as inflammatory cytokines and enzymes (TNF-α, IL-6, MCP-1, SAA) and matrix metalloproteases (Gómez-Ambrosi et al., 2006; Aggarwal, 2009). Among all these molecules, perhaps the transcription factor NF-κ B is the central mediator of inflammation (Aggarwal, 2004).

Leptin, the product of the *ob* gene, is an adipocyte-derived hormone that is a central mediator in regulating body weight by signaling the size of the adipose tissue mass (Zhang et al., 1994). Leptin levels are closely correlated with adiposity in obese rodents and humans (Maffei et al., 1995; Frühbeck et al., 1998, 2001b; Muruzábal et al., 2002). Subsequent studies have suggested that this hormone may be linked to the increased incidence of cancer in obesity (Khandekar et al., 2011). Leptin has attracted attention due to its potential function as an antiapoptotic, mitogenic, proangiogenic, and prometastatic agent, as observed in numerous *in vitro* studies (Frühbeck, 2006a; Fruhbeck, 2006b; Park et al., 2011). Circulating levels of leptin have been investigated to determine the correlation with cancer and progressive disease. A strong association between leptin levels and colorectal and endometrial cancer has been reported (Petridou et al., 2002; Koda et al., 2007a). However, the findings of clinical studies of the relationship between leptin and breast cancer are inconsistent (van Kruijsdijk et al., 2009). Interestingly, many colorectal, breast, and endometrial cancers overexpress the leptin receptor OB-R (Koda et al., 2007a,b). Leptin produced by adjacent adipose tissue might promote the growth of colorectal cancer enhancing the proliferation of colon cancer cells although other factors released by adipocytes are also likely to be involved in the process. It suggests that the presence of tumor-associated adipose tissue represents an important microenvironmental influence (Amemori et al., 2007; Vansaun, 2013).

It has now been extensively documented that adiponectin expression is inversely correlated with obesity (Scherer et al., 1995; Hu et al., 1996). Adiponectin may influence cancer risk through its well-recognized effects on insulin resistance, but it is also plausible that adiponectin acts on tumor cells directly (Yamauchi et al., 2001; Barb et al., 2007). Interestingly, several cancer cell types express the adiponectin receptors AdipoR1 and AdipoR2 that may mediate the inhibitory effects of adiponectin on cellular proliferation (Kim et al., 2010). Epidemiologic studies show that low levels of adiponectin have an inverse association with the risk for the development of multiple cancers as well as advanced progression of disease (Wei et al., 2005; Barb et al., 2007; Bao et al., 2013). In a prospective analysis, adiponectin levels were inversely associated with endometrial (Dal Maso et al., 2004) and breast cancer risk in postmenopausal women (Tworoger et al., 2007). Adiponectin also inhibits prostate and colon cancer cell growth (Bub et al., 2006). In a mouse tumor model, adiponectin markedly induced a cascade activation of caspase−8, −9, and −3, which leads to cell death inhibiting primary tumor growth (Brakenhielm et al., 2004b).

TNF-α, a cytokine originally identified as mediating endotoxin-induced tumor necrosis (Carswell et al., 1975), has been shown to be involved in the development of a number of cancers through the promotion of vessel growth and tumor destruction by direct cytotoxicity angiogenesis (Leibovich et al., 1987) as well as the metastatic potential of circulating tumor cells (Orosz et al., 1993). However, although TNF-α is the most potent activator of NF-κ B, elevated levels of TNF-α in tissue or serum are not very common in cancer patients (Aggarwal and Gehlot, 2009). The increased circulating levels of TNF-α of both obese rodents and obese humans, suggest a possible link between obesity and tumorigenesis (Khandekar et al., 2011). In this regard, obesity-promoted hepatocellular carcinoma development was dependent on increased production of the cytokines TNF-α and IL-6, which cause hepatic inflammation and activation of the oncogenic transcription factor STAT3 (Park et al., 2010). Diet-induced obesity produces an elevation in colonic TNF-α giving rise to a number of alterations including the dysregulation of the Wnt signaling pathway, with an important involvement in colorectal cancer (Liu et al., 2012).

Another pro-inflammatory molecule produced in adipose tissue is IL-6. The circulating levels of IL-6 are higher in subjects with obesity-related insulin resistance (Kern et al., 2001). IL-6 is a pleiotropic cytokine with a significant role in growth and differentiation (Ghosh and Ashcraft, 2013) that signals to the nucleus through STAT3, an oncoprotein that is activated in many human cancers and transformed cell lines (Bromberg et al., 1999). Interestingly, STAT3 is activated by leptin (Vaisse et al., 1996) and probably may have a role in the pro-tumorigenic effects of this adipokine. Moreover, different studies indicate that serum IL-6 levels are a negative indicator of the development of breast cancer in overweight or obese patients with prominent insulin resistance (Gonullu et al., 2005; Knupfer and Preiss, 2007).

MCP-1 is a member of the CC chemokine superfamily (Panee, 2012) that plays a crucial role in recruitment and activation of monocytes during acute inflammation and angiogenesis (Charo and Taubman, 2004). Circulating levels of MCP-1 are generally increased in obese patients compared to lean controls (Catalán et al., 2007). Gene expression levels in adipose tissue follow the same trend, being higher in the visceral and subcutaneous adipose tissue of obese patients compared to lean volunteers (Huber et al., 2008). There is emerging evidence that MCP-1 induces tumor cell proliferation via activation of the phosphatidylinositol 3-kinase/protein kinase B (PI3K/Akt) pathway in various cancer types (Loberg et al., 2006). Moreover, MCP-1 promotes cancer tumorigenesis indirectly via its effects on macrophage infiltration (Walter et al., 1991). It has been described that MCP-1 is highly expressed by breast tumor cells and has causative roles in breast malignancy and metastasis (Soria and Ben-Baruch, 2008). The pleiotropic roles of CCL2 in the development of cancer are mediated through its receptor, CCR2 (Lu et al., 2007).

Novel adipokines involved in obesity-associated inflammation have emerged as important players of tumor growth (Catalán et al., 2011d). OPN is a secreted glycoprotein expressed by different cellular types (Brown et al., 1992). Recently, several studies have highlighted the expression of OPN in adipose tissue of both humans and mice and its involvement in obesity and obesity-associated T2D promoting inflammation and the accumulation of macrophages in adipose tissue (Gómez-Ambrosi et al., 2007; Nomiyama et al., 2007). High OPN expression in the primary tumor is associated with early metastasis and poor outcome in human breast and other cancers (Denhardt et al., 2001). LCN-2 also known as neutrophil gelatinase associated lipocalin is a component of the innate immune system with a key role in the acute-phase response to infection (Flo et al., 2004). Increased levels of LCN-2 in visceral adipose tissue in human obesity and a relationship with pro-inflammatory markers has also been described (Catalán et al., 2009, 2013). In addition to inhibiting invasion and metastasis, LCN-2 also appears to be a negative regulator of angiogenesis in cancer cells (Chakraborty et al., 2012). Tenascin-C (TNC) is an extracellular matrix glycoprotein specifically induced during acute inflammation and persistently expressed in chronic inflammation (Chiquet-Ehrismann and Chiquet, 2003; Udalova et al., 2011). Increased expression of TNC has been described in most solid cancers, playing important roles in enhancing proliferation, invasion and angiogenesis during tumorigenesis and metastasis (Midwood and Orend, 2009; Midwood et al., 2011). In this line, elevated expression levels of TNC have been found in visceral adipose tissue in obesity with a tight association of genes being involved in maintaining the chronic inflammatory response associated to obesity (Catalán et al., 2011c). YKL-40 is another adipokine involved in inflammation and cancer cell proliferation. YKL-40 is a growth factor with elevated gene and protein expression levels in visceral adipose tissue in human obesity-associated T2D (Catalán et al., 2011b). Moreover, circulating levels of this cytokine are described as an obesity-independent marker of T2D (Nielsen et al., 2008). On the other hand, elevated levels of YKL-40 were found in patients with different types of solid tumors, including several types of adenocarcinomas, small cell lung carcinoma, glioblastoma, and melanoma (Johansen et al., 2006). Calprotectin is a member of the S100 protein family released by activated phagocytes and recognized by TLR4 on monocytes (Vogl et al., 2007). Calprotectin is not only involved in differentiation and cell migration but has also been identified as an important regulator of inflammation in cancer development and tumor spreading (Hiratsuka et al., 2008; Ehrchen et al., 2009). The increased levels of calprotectin in obesity and obesity-associated T2D have been shown decrease after weight loss achieved by RYGB (Catalán et al., 2011a).

## Conclusions

**Figure 2:**
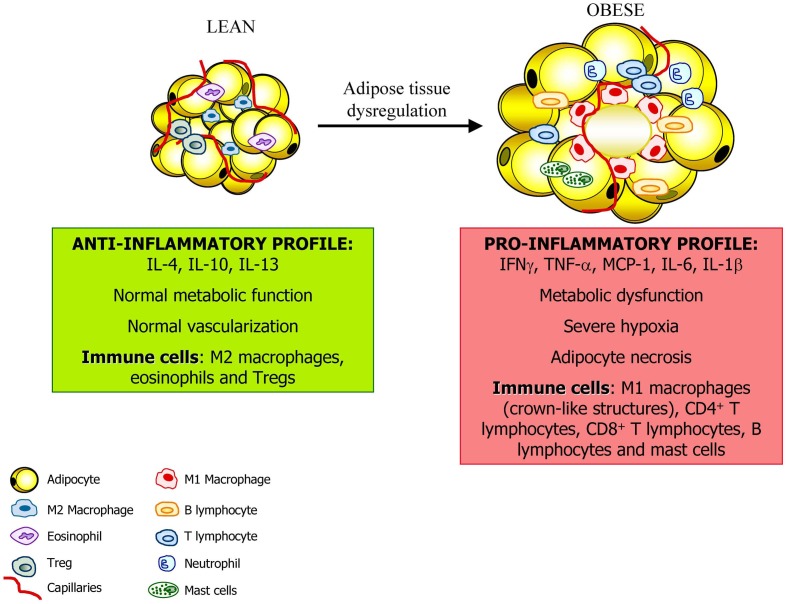
**Adipose tissue constitutes an active endocrine organ.** In the lean state, adipose tissue exhibits resident macrophages polarized toward an M2 status and Treg cells involved in support a metabolic homeostasis. Moreover, the inflammation is controlled through the eosinophil-derived interleukin (IL)-4 and IL-13 as well as the IL-10 secreted by Treg cells and M2 macrophages. With a progression of obesity, adipocytes undergo hypertrophy and release adipokines that promotes the acquisition of an M1 macrophage phenotype with increased production of pro-inflammatory factors such as tumor necrosis factor-α, (TNF-α), monocyte chemotactic protein (MCP)-1, and IL-6. This is accompanied by the infiltration of mast cells and T lymphocytes contributing to the dysregulation of adipose tissue and favoring and perpetuating an inflammatory state.

The prevalence of obesity has risen steadily for the past several decades. Excess of adiposity is associated with increased death rates for all cancers combined and for cancers at multiple specific sites with the strongest evidence for endometrial cancer, postmenopausal breast cancer, colon cancer, renal cell carcinoma of the kidney, liver, gallbladder, esophageal, and pancreatic cancer. The mechanisms linking obesity and cancer are unclear but low-grade chronic inflammation, dysregulation of growth signaling pathways, chronic hyperinsulinemia, and hypoxia associated to obesity are widely accepted as important factors in cancer pathogenesis. Particular attention is placed on the pro-inflammatory environment associated with the obese state, specifically highlighting the involvement of infiltrated immune cells into adipose tissue. In this sense, the understanding of the regulatory mechanisms that lead to polarization of macrophages or lympocytes in adipose tissue toward a pro-inflammatory phenotype will provide new ways to control adipose tissue inflammation (Figure 2). A better understanding of the mechanistic links between obesity and cancer will help to identify intervention targets and strategies to avoid the pro-tumorigenic effects of obesity.

### Conflict of interest statement

The authors declare that the research was conducted in the absence of any commercial or financial relationships that could be construed as a potential conflict of interest.
